# Research on the Impact of Industrial Policy on the Innovation Behavior of Strategic Emerging Industries

**DOI:** 10.3390/bs14040346

**Published:** 2024-04-22

**Authors:** Wei Yang, Xueke Wang, Dandan Zhou

**Affiliations:** School of Economics and Management, Shanghai Ocean University, Shanghai 201306, China; wyang@shou.edu.cn (W.Y.); wangxueke139@163.com (X.W.)

**Keywords:** industrial policy, strategic emerging industries, innovative behavior, government subsidies, tax incentives

## Abstract

Cultivating strategic emerging industries (SEIs) is an important strategy for most countries around the world to seize the economic frontier. Academics have not yet reached a unified conclusion on whether the adoption of industrial policy from the government level can effectively promote its R&D and innovation behaviors and contribute to industrial upgrading. Based on the data regarding 33,425 Shanghai and Shenzhen A-share-listed companies from 2007 to 2020, this article employs the difference-in-difference model (DID) and the mediated effect model to identify the effect and mechanism of how industrial policy affects the innovation behavior of SEIs. The results of this study show that the promulgation and implementation of industrial policies can help stimulate enterprises to carry out R&D and innovation behaviors and improve the innovation level of SEIs. Its promoting effect on state-owned enterprises is more significant than that on non-state-owned enterprises, and its promoting effect on the eastern and central regions is more significant than that on the western region. Further analysis reveals that government subsidies and tax incentives are important transmission mechanisms through which industrial policy affects firms’ innovation, with government subsidies playing a positive facilitating role and tax incentives having a negative impact.

## 1. Introduction

Industrial policy is an important regulatory tool for the government to promote the effective allocation of resources and the sound development of industries [1]. Economies develop differently at each stage, and the focus of industrial policy implementation is skewed. In October 2010, the State Council issued the Decision on Accelerating the Cultivation and Development of Strategic Emerging Industries (hereinafter referred to as the Decision), announcing that strategic emerging industries (hereinafter referred to as SEIs) would be a key area for future economic and social development. Since then, China’s central and local governments have issued a series of relevant policies, such as adopting fiscal and tax support policies, increasing credit support, and setting up special funds, etc., to guide and plan the development of SEIs in a more clear and detailed manner [2]. Indeed, industrial policy has a long history of being used by many countries to support the development of SEIs [3] and to stimulate economic growth [4]. China is in a period of the transformation of the mode of economic development; at this time, this occurs through a variety of direct and indirect interventions to improve the market competition environment. Seizing opportunities for the development of SEIs is an important issue faced by Chinese government [5].

Innovation is the catalyst for industrial upgrading [6] and can help institutions to achieve the expected economic gains [7]. At the beginning of its proposal, the industrial policy related to SEIs emphasized that the key to its development lies in stimulating innovative behaviors, enhancing the independent innovation capability of the whole industrial chain, breaking through the core technology with the power of industrial policy, and improving the competitiveness of the industry [8]. The survey report of the Ministry of Information Industry on the typical enterprises of SEIs shows that under the role of a series of policies to stabilize growth, SEIs have gained sustained development by playing a role in innovation drive. However, in the academic world, there has not been a unified conclusion on the effectiveness of the implementation of industrial policy. Some scholars believe that industrial policy can help firms increase funding, reduce market risk, improve productivity [9], fuel firm innovation [10,11], and promote economic growth [12]; some scholars also point out that industrial policy has caused the generation of pseudo-R&D behavior through fiscal support [13], promoted the quantity rather than the quality of firm innovation [14], and only promoted innovation in high-tech industries while hindering the economic performance of low-tech industries [15].

A favorable environment for innovation and entrepreneurship requires the government to take the lead in creating it. Has the Chinese government’s use of government subsidies, tax incentives, and other measures truly promoted substantive innovation behaviors among enterprises and contributed to the sustainable and healthy development of SEIs? Based on the above background, this paper intends to address the following questions: (1) What is the impact of industrial policy on the innovation level of SEIs? (2) Through which mechanism does industrial policy affect the innovation level of enterprises, and are the effects of different mechanisms the same? (3) What adjustments need to be made in the implementation of industrial policy in China to produce the best effect?

Innovation is the soul of the development of SEIs [16]. In the context of the new normal of economic development, it is of great policy significance to study how industrial policy affects the innovation of SEIs, how to organically combine major scientific and technological breakthroughs with emerging social needs, and how to reasonably and appropriately implement industrial policy to promote the healthy development of SEIs [17]. Based on this, this paper selects the panel data of A-share-listed companies from 2007 to 2020 as the research samples, empirically examines the impact of industrial policy on the innovation of SEIs by using the difference-in-difference model (DID), and further explores the specific mechanism of the role of industrial policy by utilizing the mediation effect model.

The possible marginal contributions of this paper are as follows: first, combining relevant authoritative documents to establish a database of A-share-listed companies in SEIs, sorting out SEIs as research samples, and purposefully examining the incentive effects of macro national policies on the innovation behaviors of micro enterprises; second, based on the background of China’s uneven regional development and the long-term coexistence of state-owned and private firms in the market, a detailed heterogeneity analysis is conducted according to the nature of property rights and location, scientifically identifying the differences in firms for which industrial policy functions as an incentive for innovation; third, unlike previous studies that used a single policy tool, this paper comprehensively analyzes the two support strategies of ex ante incentives, government subsidies, and ex post incentives, tax preferences, revealing the actual path and impact effects of industrial policy.

## 2. Theoretical Analysis and Research Hypothesis

### 2.1. Promotional Effect of Industrial Policy on Innovation in SEIs

The introduction of industrial policy was initially associated with the neoclassical school of “market failure” [18]. Neoclassical economics views market failure as brought about by imperfections in market functions such as monopoly, externalities, public goods, and information asymmetry. Specifically the constraints are as follows: first, there is a positive externality in the innovation activities of SEIs, i.e., innovation research and development is expensive, but replicating the results of innovation is cheap; second, the absence of a market for public goods is a characteristic of public goods, which producers are reluctant to produce but which are genuinely needed for social development, and, therefore, “free-riding” behavior cannot be avoided [19]; third, there is information asymmetry between enterprises and governments as subjects in market economic activities, which can lead to moral hazard, principal–agent, and other problems [20]. When the market fails, in order to maximize the efficiency of resource allocation, it is necessary to resort to government intervention, and the theory of market failure provides the basis for the legitimacy of government intervention [21].

Industrial policy can protect industries that are currently weak but have high technological outgrowth and where production costs diminish as inputs increase [22]. SEIs are characterized by high risk, high investment, and long R&D cycles [23], and innovation products have obvious public goods attributes. Relying only on the market mechanism may not be able to realize the optimal allocation, because the phenomenon of market failure exists from time to time, i.e., the market economy itself cannot effectively allocate resources. Therefore, interventionism in industrial policy is crucial [24]. Reasonable industrial policies can make up for the inherent defects of the market mechanism, enhance the productivity of enterprises, and promote economic development [25,26]. Specifically, the positive impact of industrial policy is manifested in the following aspects: first, easing the financing constraints of enterprises. Innovation requires large amounts of capital and talent, and the shortage of resources and costs is often an important constraint on innovation. Industrial policy can reduce the risk of innovation by subsidizing enterprises through government subsidies and tax incentives, thereby alleviating financing constraints in the short term [27]. Second, firms are incentivized to compete in R&D. Supportive industrial policies, such as industry deregulation, will lead to the more frequent entry and exit of firms from the market, and competition for resources between old and new firms will intensify competition in the industry. The mechanism of survival of the fittest will prompt enterprises to take the initiative to increase their R&D and innovation efforts in order to enhance their competitive advantages. Even for the purpose of responding to policies to obtain financial and tax support, enterprises will increase their innovation activities [28]. Finally, it helps enterprises to send positive signals to the market. A directory of supported industries can send clear positive signals to the market that they have greater development potential and better prospects. As a result, policy-supported firms are more likely to be favored by investors, thus helping to attract resources for R&D investment [29]. Based on the above analysis, the following hypothesis is proposed:

**Hypothesis** **1.** *There is a facilitating effect of industrial policy on innovation in SEIs.*

### 2.2. Heterogeneous Impact of Industrial Policy on Innovation in SEIs

No policy can be universal [30]. Industrial policy is formulated by the government to reduce costs, improve efficiency, and provide a favorable environment for enterprise innovation; however, its implementation will be affected by many factors such as the enterprise itself, the market environment, and the economic situation. From the enterprise level, state-owned enterprises (SOEs) are, to a certain extent, more closely linked to the government, and it is easier for them to obtain policy subsidies from the state, so that they can better carry out R&D and innovation; private enterprises tend to spend a great deal of time and effort in order to obtain government subsidies, and the resulting costs will have a certain crowding-out effect on the R&D investment of the enterprise [2]. At the regional level, the ability of industrial policy to drive the technological innovation activities of firms in the region is influenced by the political and economic incentives of local governments in the process of industrial policy formulation and implementation [31]. Economically developed regions, incentivized by industrial policy, tend to pursue innovative projects that promote long-term development. However, Fang et al. (2020) also point out that local government subsidies may contribute to the creation of zombie firms [32]. Especially for economically underdeveloped regions, industrial policy support for SEIs may induce rent-seeking corruption, leading to resource misallocation. Specifically, government officials at all levels will use the power they hold over the approval of financial projects and the use of funds to find opportunities for corruption and to realize their own rent-seeking activities [33]. Based on the above analysis, the following hypothesis is proposed:

**Hypothesis** **2.** *There is property rights heterogeneity and regional heterogeneity in the impact of industrial policy on innovation in SEIs.*

### 2.3. The Influence Mechanism of Industrial Policies on Innovation in SEIs

Government subsidies and tax incentives are the main fiscal incentive tools of industrial policy, with the former being a supply-side industrial policy and the latter a demand-side policy tool [34]. Government subsidies, as an important financial tool to support enterprise innovation, can alleviate the financing difficulties of enterprises and make it easier for them to obtain external funds, so as to increase the R&D investment in innovation activities [35], which belongs to ex ante incentives. Tax incentives are mainly ex post incentives through the implementation of tax exemptions, tax rebates, and additional deductions for R&D expenses to reduce the marginal cost of innovation for firms [36] and to help firms accumulate internal funds so that they have the ability to compete for innovation projects in the market [26]. Both tax incentives and government subsidies can increase firms’ investment in innovative R&D, but there are differences between the two in terms of incentive methods, implementation costs, and incentive effects [37]. Overall, through the above two channels, the release and implementation of industrial policy can convey positive signals to firms and the market, which can have an impact on firms’ innovative behavior [38]. Based on the above analysis, the following hypotheses are proposed:

**Hypothesis** **3a.** *Industrial policy has an impact on the innovation of SEIs through government subsidies*.

**Hypothesis** **3b.** *Industrial policy has an impact on the innovation of SEIs through tax incentives.*

## 3. Research Design

### 3.1. Sample Selection and Data Source

China has not yet formed a unified catalog of SEIs (See Supplementary Materials for specific definitions and examples of SEIs), and the most authoritative and mainstream documents on SEIs are as follows: the Guidance Catalogue of Key Products and Services for Strategic Emerging Industries compiled by the State Council, the Classification of Strategic Emerging Industries (2012) (Trial), and the Classification of Strategic Emerging Industries (2018) issued by the National Bureau of Statistics (NBS). However, the above categorization standards have not been refined to the micro-enterprise level; therefore, this paper combines the CSRC’s Guidelines on Industry Classification of Listed Companies (revised in 2012) to establish a database of A-share-listed companies in SEIs and identifies 20 of the major categories as SEIs.

This paper takes the A-share-listed companies in Shanghai and Shenzhen from 2007 to 2020 as the research samples, deletes the samples of financial, ST, *ST, and companies with missing values, and finally obtains 33,425 observations. Among them, there are 13,612 samples belonging to listed companies in SEIs and 19,813 samples of other listed companies. The data on the fundamental indicators of enterprises including enterprise size, gearing ratio, nature of property rights, government subsidies, and tax incentives are from the WIND database, and the data on the company’s patent applications are from the CNRDS database. To eliminate the effect of outliers on the test results, the 1% and 99% percentiles of the continuous variables were Winsorized and the following empirical reports are based on the processed data results.

### 3.2. Measurement Model

The difference-in-difference model (DID), a common method for assessing the effects of policies, can largely avoid the endogeneity problem [39]. Therefore, to test the effect of industrial policy on corporate innovation, this paper constructs a difference-in-difference (DID) model by setting listed companies belonging to SEIs as the experimental group and other listed companies as the control group:

(1) $$\begin{array}{c} \mathrm{Ln}P_{it}=\beta_{0}+\beta_{1}{Post}_{it}\times {Policy}_{it}+\beta_{2}{Controls}_{it}+f_{i}+f_{t}+\varepsilon_{it} \end{array}$$

where LnP_it_ measures enterprise innovation, Post and Policy are dummy variables, Controls are firm-level control variables, i denotes individual firms, t denotes time, $f_{i}$ is a firm (individual) fixed effect, $f_{t}$ is a time-fixed effect, and $\varepsilon_{it}$ denotes a random interference term.

### 3.3. Variable Definition

(1) Explained variable: firm innovation (LnP). The existing literature measures technological innovation by selecting indicators mainly from two dimensions: innovation input is mainly measured by R&D capital investment and personnel input, and innovation output is mainly measured by the number of patents and new product output value. R&D investment and new product output may be falsely reported, whereas patent data are based on factual innovations and are, therefore, more reliable [40]. The number of patents granted is affected by human factors such as government patent agencies and is prone to abnormal changes, while the number of patent applications more accurately reflects innovation performance [41]. Furthermore, among the three kinds of patents, invention patents are the most inventive patents [42], which are more representative of valuable innovative technologies. In consideration of these facts, this article adopts the number of invention patent applications plus 1 to measure enterprise innovation. Of course, we must recognize that patent application data is only one aspect of measuring innovation, and it also has limitations and does not provide a comprehensive and accurate measure of innovation. Therefore, the article also selected firms’ R&D investment indicators for a robustness test.
(2) Explanatory variables: dummy variable (Policy), a grouping variable for the difference-in-difference model, divided into experimental and control groups. Listed companies in SEIs supported by industrial policies are assigned a value of 1, and the rest of the samples are assigned a value of 0; for the dummy variable (Post), the State Council issued the article “Decision” in October 2010, which is the first official document on SEIs from the national level after the global financial crisis. In order to maintain the completeness of the policy year as well as to take into account the lag of the policy, the time shock point of the policy variable is approximated as the year 2011 after Decision was issued, and the value of Post is assigned as 0 before 2011 and 1 after 2011.
(3) Mediating variables: government subsidies, measured as the total amount of government grants received by the firm minus tax benefits returned in the period/total assets; and tax benefits, measured as each tax rebate received by the firm/(each tax rebate received + each tax paid).
(4) Control variables: considering that firms’ innovation activities are affected by their own development, this paper refers to previous literature to select firm-level control variable indicators. (1) Firm size (Size). Larger firms are often able to generate cost advantages through economies of scale, reduce R&D pressures, and improve innovation efficiency. (2) Gearing ratio (Lev). Measures the solvency of a business. (3) Return on assets (Roa). Reflects the profitability and input–output status of the enterprise. (4) Current Ratio (CR). Reflects the short-term solvency of the enterprise. In order to reduce the effect of endogeneity, the control variables are lagged by one period. These variables are summarized in Table 1.

### 3.4. Descriptive Stats

Descriptive statistics for the main variables are shown in Table 2. The mean value of LnP, which characterizes corporate innovation, is 1.688, the minimum value is 0, and the maximum value is 8.298, indicating that the number of invention patent applications fluctuates greatly among various listed companies and that there are large differences in the innovation ability of each company. The mean value of industrial policy (Policy) is 0.407, indicating that industrial policy, as the government’s main means of promoting industrial transformation and upgrading, has a relatively broad coverage of about 41%. In addition, the mean value of government subsidies is 0.443, and the maximum value is 418.996, indicating that the government subsidizes the SEIs more strongly, but the subsidies to different enterprises vary greatly. The mean value of tax incentives is 0.092, which shows that the government’s industrial support based on the tax incentive route is relatively small.

## 4. Empirical Analysis

### 4.1. Base Regression Analysis

Column (1) of Table 3 shows that the estimated coefficient of the cross-multiplier term Policy × Post is significantly positive without adding any control variables; in order to eliminate the effects of differences in policy implementation targets and policy implementation time, a two-way fixed effects model is set up, and the regression results are shown in column (2), with the coefficient of Policy × Post still significantly positive; Column (3) adds firm-level control variables, and the regression results are basically consistent with the forefront. These results indicate that the industrial policy shock stimulates the enterprise innovation behavior in SEIs, and this empirical conclusion supports Hypothesis 1.

In terms of control variables, the estimated coefficient of firm size (Size) is positive, which shows that firm size has a positive effect on firm innovation, i.e., the larger the firm, the stronger the innovative strength. The positive coefficient of return on assets (Roa) indicates that firms with higher returns on assets are more inclined to engage in innovative activities, thus favoring higher levels of innovation. The following robustness test also verifies this hypothesis. The impacts of coefficients on the gearing ratio (Lev) and the current ratio (CR) are negative, but the estimated coefficients are both relatively small, suggesting that there may be a small and insignificant effect on firm innovation.

### 4.2. Robustness Testing

#### 4.2.1. Parallel Trend Testing

The premise of the estimation validity of the difference-in-difference model is that the changes in the experimental and control groups before being subjected to the policy shock should conform to the common trend assumption. In this paper, the common trend hypothesis is tested by setting up a dynamic effects model (as shown in Equation (2)), taking 2011 as the midpoint of the policy shock time and moving the policy window forward and backward for regression, respectively, to examine the effects of industrial policy shocks on the current period of policy implementation as well as after implementation.

(2) $$\begin{array}{c} \mathrm{Ln}P_{it}=\beta_{0}+{\sum_{2007}^{2020}\beta_{1}\mathrm{Post}}_{\mathrm{it}}\times {\mathrm{Policy}}_{\mathrm{it}}+\sum \beta_{x}{\mathrm{Controls}}_{\mathrm{it}}+f_{i}+f_{t}+\varepsilon_{\mathrm{it}} \end{array}$$

**Figure 1:**
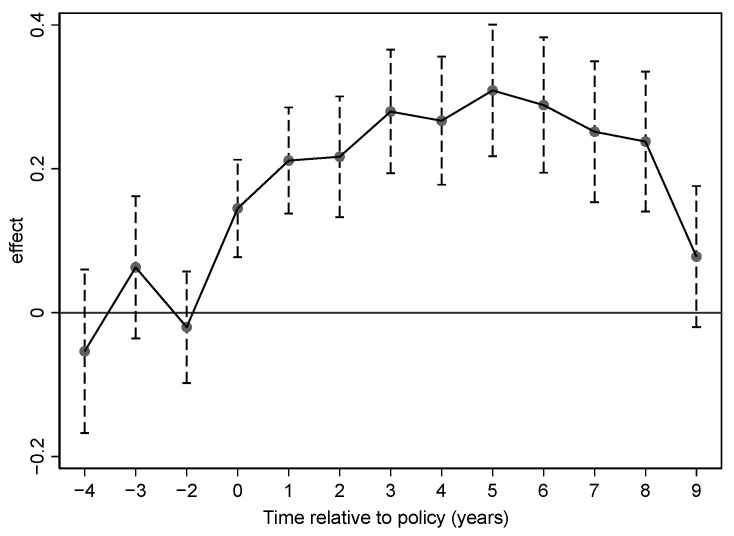
Parallel trend testing.

As shown in Figure 1, the regression coefficients of the years before the industrial policy shock in 2011 fluctuate around the 0 axis, the coefficients of the year of the policy shock are significantly positive, and the regression coefficients increase in the five years after the implementation of the policy and are significantly non-zero. Therefore, this figure shows that the hypothesis of the common trend of the sample data is valid. This step verifies the rationality and feasibility of using a difference-in-difference model in this paper.

#### 4.2.2. Placebo Testing

**Figure 2:**
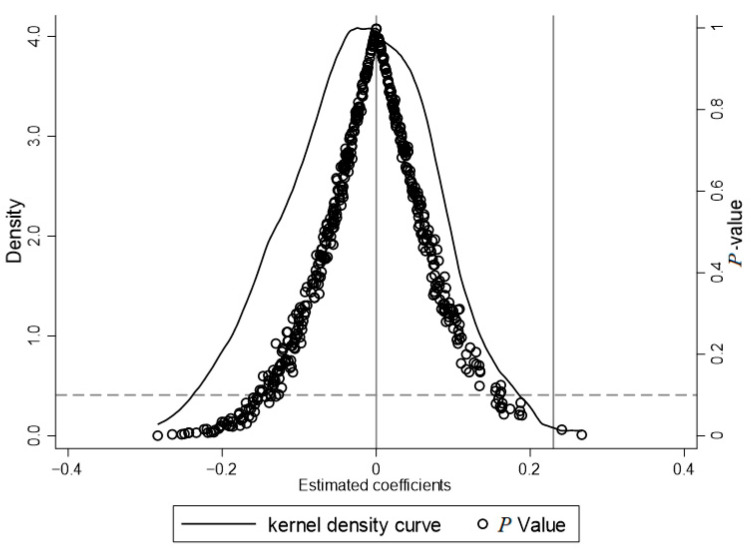
Placebo testing.

In order to further verify that the increase in the innovation level of firms in SEIs is caused by specific industrial policies rather than being influenced by other policies or randomness, this paper randomly selects 100 individuals from the sample as the experimental group and the rest of the firms as the control group for the placebo test, which is unfolded using 500 randomized trials. Figure 2 shows the distribution of DID coefficients, and the results show that the average coefficient of regression after 500 repetitions is much smaller than the original regression coefficient, and the estimated coefficients are distributed near 0 and obey the normal distribution, indicating that the results in this paper are not disturbed by other non-observed factors, and the results are robust.

#### 4.2.3. Substitution of Explanatory Variables

In order to test whether the above empirical findings are reliable, this paper utilizes alternative indicators to measure enterprise innovation and conducts robustness tests. The alternative indicator (RD) is expressed as the ratio of the amount of R&D investment from the WIND database to the operating income of listed companies. The results are shown in Table 4, which are largely consistent with the benchmark regression, again verifying that the empirical model of this paper is robust, and the results are reliable.

#### 4.2.4. Adjustment of Sample Period

The sample period selected for this paper is 2007–2020, which is adjusted to 2007–2015 in order to exclude the influence of other policies released in 2016 and later, and to accurately verify that the increase in the level of corporate innovation in SEIs is caused by a specific industrial policy in 2011. The regression results are shown in Table 5, and the coefficient of the cross-multiplier term Policy × Post is still significantly positive, which to some extent proves that the regression in this paper is robust.

### 4.3. Heterogeneity Analysis

#### 4.3.1. Heterogeneity Analysis Based on the Nature of Property Rights

Differences in the intensity of industrial policy support received by firms of different ownership may lead to differences in firms’ innovation behaviors. Specifically, state-owned enterprises (SOEs) and non-state-owned enterprises (non-SOEs) have irreplaceable roles in the national economy, but state-owned enterprises are prone to obtain more policy inclinations because of the intervention of government factors; however, non-state-owned enterprises are facing a relatively brutal market competition environment and receive limited government support. Therefore, this paper further performs a group regression on the sample of SOEs and the sample of non-SOEs and obtains the following results.

Table 6 shows that the estimated coefficients of the cross-multiplier term Policy × Post are significantly positive in both the SOE sample and the non-SOE sample and are larger in SOEs than in non-SOEs, which verifies that industrial policy can promote enterprise innovation and is more likely to increase the level of innovation in SOEs. This result may be due to the fact that SOEs receive more policy incentives than non-SOEs because of their size and political advantages; therefore, SOEs have more R&D investment to carry out innovative activities.

#### 4.3.2. Heterogeneity Analysis Based on Different Regions

Although industrial policies are uniformly promulgated by the central government, their implementation by local governments in different regions depends on their determination and efficiency in realizing industrial transformation [43]. Moreover, as resource endowments differ from region to region, the impact of industrial policies may vary in effect due to regional heterogeneity. Therefore, in this paper, according to the classification method of the National Bureau of Statistics, the provinces and cities where the enterprises are located are divided into three groups, eastern, central, and western regions, and group regression analysis is carried out.

As shown in columns (3)–(5) of Table 6, the estimated coefficients of the cross-multiplier term Post × Policy are 0.141, 0.451, and 0.106 for the eastern, central, and western regions, respectively, where only the western region is not significant and minimal. This empirical result shows that industrial policy can significantly stimulate the innovation behavior of enterprises in the eastern and central regions, with the largest positive effect on enterprises in the central region. This may be due to the central government’s emphasis on coordinated regional development and increased financial support for the central region, which has led to a more pronounced industrial policy support effect in the central region. In addition, the eastern region, which occupies the majority of resources, takes the lead in the research and development and application of technology to provide reference for the development of other regions, and the spillover and dissemination effect of this technology further promotes the development of SEIs in the central region. However, there is not enough evidence to show that industrial policy has an impact on the innovation behavior of SEIs in the western region, which may be due to the imperfect development of infrastructure and the scarcity of resources in the western region, which limits the development of its SEIs.

## 5. Impact Mechanism Test—Government Subsidies and Tax Incentives Perspective

As mentioned in the previous theoretical analysis, industrial policy intervenes in the economy by a variety of means, and government subsidies and tax incentives are two major fiscal incentive tools. This paper selects two mechanisms of government subsidies and tax incentives to further analyze the effects of industrial policy and explore the specific path of industrial policy implementation.

### 5.1. Government Subsidy Channels

In order to verify whether industrial policy can affect enterprise innovation through government subsidies, the mediation effect model is used as a test. The model is as follows:

(3) $$\begin{array}{c} {Subsidies}_{it}=\beta_{0}+\beta_{1}{Post}_{it}\times {Policy}_{it}+\beta_{2}{Controls}_{it}+f_{i}+f_{t}+\varepsilon_{it} \end{array}$$

(4) $$\begin{array}{c} \mathrm{Ln}P_{it}=\beta_{0}+\beta_{1}{Post}_{it}\times {Policy}_{it}+\beta_{2}\mathrm{Subsidies}+\beta_{3}{Controls}_{it}+f_{i}+f_{t}+\varepsilon_{it} \end{array}$$

where Subsidies = total government subsidies received by the firm minus the current tax benefits refunded/total assets.

The regression results are shown in column (1) and column (2) in Table 7. Specifically, it can be seen that the coefficient of Policy × Post on government subsidies in the results of Column (1) in Table 7 is 0.285, indicating that industrial policy shocks can positively contribute to the increase in government subsidies. According to the results of column (2), the direct effect coefficient of Policy × Post on enterprise innovation is 0.229, while the regression coefficient of government subsidy on enterprise innovation is significantly positive, indicating that the industrial policy can improve the enterprise innovation level through the channel of government subsidy, which verifies the previous analytical hypothesis that the SEIs, as an industry under cultivation and development, urgently need government support and guidance.

### 5.2. Tax Incentive Channels

In order to verify whether industrial policy can affect the innovation level of enterprises through tax incentives, the mediation effect model is used as a test. The following model is constructed:

(5) $$\begin{array}{c} {TaxPre}_{it}=\beta_{0}+\beta_{1}{Post}_{it}\times {Policy}_{it}+\beta_{2}{Controls}_{it}+f_{i}+f_{t}+\varepsilon_{it} \end{array}$$

(6) $$\begin{array}{c} LnP_{it}=\beta_{0}+\beta_{1}{Post}_{it}\times {Policy}_{it}+\beta_{2}{TaxPre}_{it}+\beta_{3}{Controls}_{it}+f_{i}+f_{t}+\varepsilon_{it} \end{array}$$

where TaxPre = each tax credit rebate received by the business/(each tax rebate received + each tax paid).

The coefficient of Policy × Post on firm innovation in the results of column (3) of Table 7 is −0.133, indicating that industrial policy shocks negatively affect the increase in tax incentives. Meanwhile, the results of column (4) show that the coefficient of the direct effect of Policy × Post on firms’ innovation is 0.225, and the regression coefficient of tax incentives on firms’ innovation is −0.020. This shows that there is a mediating effect of tax incentives between industrial policy and the innovation of SEIs, but the tax incentives have a negative moderating effect, i.e., industrial policy through the mechanism of tax incentives does not positively stimulate the innovation of SEIs. In fact, in the pre-innovation activities of SEIs, tax incentives do not provide incentives to firms without tax liabilities, and their incentive points are more slight and dispersed compared to government subsidies. In addition, possibly due to information asymmetry and differences in the objectives of the government and firms, firms do not invest the funds in innovative R&D after receiving the tax rebates but use them for profit distribution, i.e., camouflage R&D behavior [29]. This use of funds may allow the tax incentive mechanism to have a reverse disincentive effect on firms’ innovation. The results of this study are in agreement with Romero-Jordan et al. (2014) [3].

## 6. Conclusions and Policy Recommendations

### 6.1. Conclusions

This paper conducts an empirical study based on the fundamental data and invention patent application data of China’s A-share-listed companies from 2007 to 2020 to test the effect of industrial policy on innovation in SEIs from the perspective of the innovation output of micro-enterprises. The study finds the following:

The fact that industrial policy can significantly increase the number of invention patent applications by enterprises suggests that industrial policy helps to promote substantive innovation in SEIs. Heterogeneity analysis according to the grouping of the nature of property rights reveals that the innovation incentive effect of industrial policy on both state-owned and non-state-owned enterprises is significantly positive, but the promotional effect of industrial policy on state-owned enterprises is stronger. According to regional grouping, it is found that industrial policy can significantly promote enterprise innovation in the eastern region and the central region, in which the positive effect on enterprises in the central region is the largest, but the positive impact of industrial policy on the innovation activities of SEIs in the western region needs to be further examined.

This paper further explores the specific role of industrial policy to produce the effect of the path, selecting government subsidies and tax incentives as the two mechanisms for the mediation effect test. The results found that government subsidies play a significant mediating effect in the industrial policy and innovation activities of SEIs, and the increase in government subsidies positively affects the output of innovation activities of enterprises, while tax incentives have a reverse inhibitory effect on the innovation activities of SEIs, and the mediating effect is not obvious. This may be due to the irrationality and incomplete coverage of tax incentives, such as the lack of effectiveness of ex post incentives and the formation of a similar dilemma with the “European paradox”. But, of course, this does not rule out the selection of indicators related to this paper.

### 6.2. Policy Recommendations

In order to maximize market-regulating role of industrial policy, the following recommendations are made:

First, increase the support of industrial policy for SEIs. Through the previous empirical test, it can be seen that industrial policy as a whole has a facilitating effect on the innovation of SEIs. Therefore, both governments and enterprises should emphasize the supportive role of industrial policy. The government provides timely guidance and continuously strengthens resource-based tools, such as government subsidies, social financing, and tax incentives, to ensure credit financing for SEIs and help enterprises carry out innovative activities in a sustainable manner. This allows firms to maximize the major leading and driving role of strategic emerging industries for the whole economy and society, to make the allocation of resources, especially the allocation of scarce resources, reach Pareto optimization, and to help the healthy development of SEIs.

Second, improve fiscal and tax incentive policies. (1) The implementation of government subsidies needs to be optimized, which can be achieved by establishing a special service tracking system for financial funds that better detects and supervises the destination of each government subsidy. (2) The implementation of tax incentives needs to be adjusted. On one hand, improve the communication efficiency of government functional departments, so that enterprises can obtain timely policy information related to tax incentives and reduce the problem of information asymmetry in the process of policy implementation. On the other hand, improve the preferential policies on entrepreneurial investment, angel investment, venture capital, etc., and vigorously increase the amount of tax rebates and exemptions, so as to give more comprehensive policy support to SEIs. (3) Synergies between government subsidies and tax incentives need to be improved to increase the efficiency of capital utilization and reduce the risk of information asymmetry.

Third, enhance the differentiation and precision of industrial policy implementation. Future industrial policy should fully recognize the existence of property rights attributes of strategic emerging enterprises, regional development imbalance and other differentiation, according to the time and place, and seek common ground to formulate and implement industrial policy. In terms of the nature of property rights, it is necessary to alleviate the pressure on innovation resources and cost inputs of non-state enterprises, optimize the structure of property rights and investment, and eliminate market discrimination. From the perspective of regional development, it is necessary to strengthen the supply of resources, capital, technology, and other policies in favor of the less developed regions and to issue preferential policies to attract the trans-regional mobility of advanced talents and enterprises, so as to drive the industrial upgrading and innovative development of the central and western regions.

Fourth, improve inclusive and prudent supervision mechanisms. In the process of promoting the continuous innovation and upgrading of SEIs, in order to reduce the occurrence of the phenomenon of “rent-seeking and corruption” and to avoid the use of funds for low-level duplicated construction and homogenized and disorderly competition, the government should strengthen the supervision of industrial policy, ensure the fairness of policy implementation, and provide a good market environment for the innovative activities of enterprises. Only by improving the regulatory system in the operation of the market economy and reducing the information asymmetry in the market can we reduce the generation of strategic innovation and promote the further development of the substantive innovation in China’s SEIs.

## Figures and Tables

**Table 1:** Definition of variables.

Type	Name	Symbol	Definition
Explained variable	Firm innovation	LnP	Natural logarithm of the number of patent applications for inventions plus 1
Explanatory variables	DID	Policy × Post	If the enterprise belongs to the experimental group, Policy is assigned as 1, otherwise, it is assigned as 0; before 2011, Post is assigned as 0, otherwise, it is assigned as 1
Mediating variables	Government subsidies	Subsidies	Total government grants less current tax benefits refunded/total assets
	Tax benefits	TaxPre	Refunds of taxes and fees/(Refunds of taxes and fees + taxes and fees paid)
Control variables	Firm size	Size	Natural logarithm of the company’s total assets, lagged by one period
	Gearing ratio	Lev	Ratio of total liabilities to total assets, lagged by one period
	Return on assets	Roa	EBITDA to average total assets, lagged by one period
	Current ratio	CR	Ratio of total current assets to total current liabilities, lagged by one period

**Table 2:** Results of descriptive statistics.

Variant	Sample Size	Mean	Standard Deviation	Min	Max
LnP	33,425	1.688	1.528	0	8.298
Policy	33,425	0.407	0.491	0	1
Policy × Post	33,425	0.350	0.477	0	1
Subsidies	33,423	0.443	2.564	−3.932	418.996
TaxPre	33,411	0.092	1.307	−1.313	76.869
Size	33,423	3.623	1.310	1.031	7.664
Lev	33,423	0.431	0.214	0.050	0.965
Roa	33,425	0.062	0.070	−0.220	0.284
CR	33,421	2.511	2.748	0.253	17.805

**Table 3:** The impact of industrial policy on innovation in SEIs: benchmark regression results.

	(1)	(2)	(3)
	LnP	LnP	LnP
Policy × Post	0.936 ***	0.245 ***	0.230 ***
	(0.021)	(0.050)	(0.047)
Size			0.380 ***
			(0.024)
Leverage			−0.010
			(0.090)
Roa			0.020
			(0.109)
CR			−0.009 **
			(0.004)
Constant	1.348 ***	0.718 ***	−0.242 ***
	(0.021)	(0.030)	(0.082)
Firm	No	Yes	Yes
Year	No	Yes	Yes
Adjust-R^2^	0.059	0.232	0.273
N	33,425	33,425	33,421

Note: **, and *** denote significance levels of 5%, and 1%, respectively, with robust standard errors in parentheses.

**Table 4:** Measuring innovation capacity using proxy indicators.

	(1)	(2)	(3)
	RD	RD	RD
Policy × Post	2.907 ***	2.101 ***	2.102 ***
	(0.073)	(0.171)	(0.174)
Size			0.160 **
			(0.072)
Lev			−1.348 ***
			(0.349)
Roa			−5.083 ***
			(0.457)
CR			0.056 **
			(0.028)
Constant	61.099	8.323 ***	8.799 ***
	(49.381)	(0.132)	(0.265)
Firm	No	Yes	Yes
Year	No	Yes	Yes
Adjust-R^2^	0.051	0.079	0.092
N	33,392	33,392	33,389

Note: **, and *** denote significance levels of 5%, and 1%, respectively, with robust standard errors in parentheses.

**Table 5:** Adjusting the sample period to re-run the regression.

	(1)	(2)	(3)
	LnP	LnP	LnP
Policy × Post	0.748 ***	0.221 ***	0.218 ***
	(0.021)	(0.044)	(0.043)
Size			0.271 ***
			(0.030)
Lev			−0.001
			(0.001)
ROA			0.001
			(0.001)
CR			−0.018 ***
			(0.005)
Constant	1.157 ***	0.737 ***	0.057
	(0.024)	(0.025)	(0.100)
Firm	No	Yes	Yes
Year	No	Yes	Yes
Adjust-R^2^	0.056	0.189	0.209
N	17,069	17,069	17,065

Note: *** denote significance levels of 1%, respectively, with robust standard errors in parentheses.

**Table 6:** Results of heterogeneity analysis.

	LnP				
	(1)	(2)	(3)	(4)	(5)
	SOEs	Non-SOEs	Eastern Region	Central Region	Western Region
Policy × Post	0.274 *** (0.075)	0.228 *** (0.059)	0.141 ** (0.059)	0.451 *** (0.105)	0.161 (0.133)
Size	0.408 ***	0.372 ***	0.407 ***	0.398 ***	0.355 ***
	(0.038)	(0.031)	(0.032)	(0.054)	(0.060)
Lev	−0.121	0.037	−0.198	0.213	0.345
	(0.154)	(0.108)	(0.122)	(0.191)	(0.212)
Roa	−0.028	−0.011	−0.101	0.517 **	−0.282
	(0.188)	(0.133)	(0.143)	(0.241)	(0.248)
CR	−0.029 ***	−0.007	−0.014 ***	−0.002	0.007
	(0.009)	(0.005)	(0.005)	(0.011)	(0.012)
Constant	−0.453 ***	−0.037	−0.096	−0.668 ***	−0.530 **
	(0.154)	(0.094)	(0.104)	(0.191)	(0.226)
Firm	Yes	Yes	Yes	Yes	Yes
Year	Yes	Yes	Yes	Yes	Yes
Adjust-R^2^	0.316	0.243	0.261	0.322	0.274
N	13,374	20,047	21,873	5568	4381

Note: **, and *** denote significance levels of 5%, and 1%, respectively, with robust standard errors in parentheses.

**Table 7:** Results of the intermediary mechanism test.

	(1)	(2)	(3)	(4)
	Subsidies	LnP	TaxPre	LnP
Policy × Post	0.285 *	0.229 ***	−0.133 **	0.225 ***
	(0.151)	(0.047)	(0.058)	(0.047)
Subsidies		0.006 *		
		(0.003)		
TaxPre				−0.020 ***
				(0.007)
Size	−0.385 **	0.382 ***	−0.023	0.383 ***
	(0.168)	(0.024)	(0.023)	(0.024)
Lev	0.416 **	−0.012	0.061	−0.004
	(0.167)	(0.090)	(0.138)	(0.090)
Roa	2.059 **	0.007	−0.415 **	0.012
	(0.873)	(0.110)	(0.185)	(0.110)
CR	0.006	−0.010 **	0.018 **	−0.009 **
	(0.008)	(0.004)	(0.008)	(0.004)
Constant	0.966 **	−0.248 ***	0.213 **	−0.253 ***
	(0.426)	(0.082)	(0.095)	(0.082)
Firm	Yes	Yes	Yes	Yes
Year	Yes	Yes	Yes	Yes
Adjust-R^2^	0.011	0.273	0.005	0.274
N	33,421	33,421	33,409	33,409

Note: *, **, and *** denote significance levels of 10%, 5%, and 1%, respectively, with robust standard errors in parentheses.

## Data Availability

Data will be made available on request.

## Supplementary Materials

The following supporting information can be downloaded at: https://www.mdpi.com/article/10.3390/bs14040346/s1, Table S1. Classification of strategic emerging industries.
