# Supplementary material for: Research on the Impact of Industrial Policy on the Innovation Behavior of Strategic Emerging Industries
Source: Behav Sci (Basel). 2024 Apr 22;14(4):346. doi: 10.3390/bs14040346 (PMC11047462; doi:10.3390/bs14040346)
Supplement: Supplementary file 1 [file behavsci-14-00346-s001.zip › behavsci-2821797-supplementary.pdf]

Supplementary Materials

This paper establishes A database of a-share listed companies in strategic emerging industries in combination with the CSRC Industry Classification Guidelines of Listed Companies (revised in 2012), and 20 categories are identified as strategic emerging industries. Specifically, including the following industries.

Petroleum processing, coking and nuclear fuel processing industry (25), chemical fuels and chemical products manufacturing industry (26), pharmaceutical manufacturing industry (27), chemical fiber manufacturing industry (28), rubber and plastic products industry (29), general-purpose equipment manufacturing industry (34), special equipment manufacturing industry (35), automobile manufacturing industry (36), railroads, ships, aerospace and Other Transportation Equipment Manufacturing Industry (37), Electrical Machinery and Equipment Manufacturing Industry (38), Computer, Communication and Other Electronic Equipment Manufacturing Industry (39), Instrument and Meter Manufacturing Industry (40), Other Manufacturing Industry (41), Waste Resource Comprehensive Utilization Industry (42); Telecommunication, Radio and Television and Satellite Transmission Services under Information Transmission, Software and Information Technology Services Industry (63), Internet and Related Services (64), Software and Information Technology Services (65); Research and Experimental Development (73), Professional and Technical Services (74) under Scientific Research and Technical Services; Ecological Protection and Environmental Governance (77) under Water Conservation, Environment, and Public Facilities Management.

Strategic emerging industry is the concept of industrial development at national level based on seizing the commanding heights of the new round of economic and technological development, and it is a composite concept. Firstly, strategic emerging industries reflect the strategic needs of the country, which are special industries scientifically selected to solve a series of major and urgent social needs and economic development needs; secondly, it is the part of emerging industries that can grow into leading industries or pillar industries, and it is a dynamic concept with the consideration of time dimension. According to the relevant documents of the National Development and Reform Commission, it can be defined as: Strategic emerging industries refer to those industries which are based on major technological breakthroughs and major development needs, have a significant leading role in the overall economic and social situation and long-term development, and are an important force to guide the future economic and social development.

According to the Classification of Strategic Emerging Industries (2018) released by the China Statistical Bureau, strategic emerging industries involve nine major fields such as new-generation information technology industry, high-end equipment manufacturing industry, new material industry and biological industry, with a total of 166 categories. The details are as follows:

Table S1. Classification of strategic emerging industries.

Classification name of category I	Classification name of category II
1 New generation of information technology industry	1.1 Next generation information network industry
	1.2 Electronic core industry
	1.3 Emerging software and new information technology services
	1.4 Internet and cloud computing, big data services
	1.5 artificial intelligence (AI)
2 High-end equipment manufacturing industry	2.1 Intelligent manufacturing equipment industry
	2.2 Aviation equipment industry
	2.3 Satellite and application industry
	2.4 Rail transit equipment industry
	2.5 Marine engineering and equipment industry
3 New materials industry	3.1 Advanced steel materials
	3.2 Advanced non-ferrous metal materials
	3.3 Advanced petrochemical and chemical industry of new materials
	3.4 Advanced, inorganic and non-metallic materials
	3.5 High-performance fiber and products and composites
	3.6 Frontier new materials
	3.7 New materials-related services
4 bioindustry	4.1 Biomedical industry
	4.2 Biomedical engineering industry
	4.3 Biological agriculture and related industries
	4.4 Biomass energy industry
	4.5 Other biological industry
5 New energy vehicle industry	5.1 New energy vehicle vehicle manufacturing
	5.2 New energy vehicle equipment, spare parts manufacturing
	5.3 Manufacturing of new energy vehicle-related facilities
	5.4 New energy vehicle-related services
6 New energy industry	6.1 Nuclear power industry
	6.2 Wind energy industry
	6.3 Solar energy industry
	6.4 Biomass energy and other new energy industries
	6.5 Smart grid industry
7 Energy conservation and environmental protection industry	7.1 High efficiency and energy saving industry
	7.2 Advanced environmental protection industry
	7.3 Resource recycling industry
8 Digital creative industry	8.1 Digital creative technology and equipment manufacturing
	8.2 Digital cultural and creative activities
	8.3 Design services
	8.4 Digital creativity and integration services
9 Related service industries	9.1 New technology and innovation and entrepreneurship services
	9.2 Other related services industries

Source: Strategic Emerging Industries Classification (2018).
